# Regeneration of *Betula albosinensis* in Strip Clearcut and Uncut Forests of the Qinling Mountains in China

**DOI:** 10.1371/journal.pone.0059375

**Published:** 2013-03-22

**Authors:** Yaoxin Guo, Gang Li, Youning Hu, Di Kang, Dexiang Wang, Gaihe Yang

**Affiliations:** 1 College of Agronomy, Northwest A&F University, Yangling, Shaanxi, China; 2 College of Life Science, Northwest A&F University, Yangling, Shaanxi, China; 3 College of Forestry, Northwest A&F University, Yangling, Shaanxi, China; DOE Pacific Northwest National Laboratory, United States of America

## Abstract

To contribute to a better understanding of the regeneration strategy of *Betula albosinensis* forests and the likely reasons behind either the successful recovery or failure after strip clearcutting, we compared the population structures and spatial patterns of *B. albosinensis* in eight *B. albosinensis* stands in Qinling Mountains, China. Four cut and four uncut stands were selected, and each sampled using a single large plot (0.25 ha). Results indicated that, on the one hand, *B. albosinensis* recruitment was scarce (average of 48 stems ha^−1^) in the uncut stands, relative to the mature population (average of 259 stems ha^−1^), suggesting a failure of recruitment. On the other hand, the subsequent regeneration approximately 50 years after the strip clearcutting showed that the density of the target species seedlings and saplings has increased significantly, and the current average density of seedlings and saplings was 156 stems ha^−1^. The clumped spatial pattern of *B. albosinensis* suggested that their regeneration was highly dependent on canopy disturbance. However, recruitment remained poor in the uncut stands because most gaps were small in scale. The successful regeneration of sunlight-loving *B. albosinensis* after strip clearcutting was attributed to the exposed land and availability of more sunlight. Bamboo density did not influence *B. albosinensis* recruitment in the uncut stands. However, stand regeneration was impeded after strip clearcutting; thus, removing bamboo is essential in improving the competitive status of *B. albosinensis* at the later stage of forest regeneration after clearcutting. The moderate severity of disturbance resulting from strip clearcutting reversed the degeneration trend of primary *B. albosinensis* stands. This outcome can help strike a balance between forest conservation and the demand for wood products by releasing space and exposing the forested land for recruitment. Life history traits and spatiotemporal disturbance magnitude are important factors to consider in implementing effective *B. albosinensis* regeneration strategies.

## Introduction

*Betula albosinensis*, a deciduous hardwood, is a tree species endemic to China, distributed in the mid-high mountains of warm temperate regions. As one of the most important species in the Qinling Mountains, *B. albosinensis* thrives over a wide elevation range of 1950 m to 2750 m [1], [2]. The community (i.e., species composition, spatial structure, and gap characteristics) [3]–[5] and seed germination characteristics [6], [7] of *B. albosinensis* forests have been studied. No direct study, however, has reported on the regeneration of *B. albosinensis* trees in forests where they dominate after natural disturbance or artificial management treatments. This type of ecological knowledge is essential in implementing conservation strategies and ensuring the sustainable utilization of forests [8], [9].

Tree regeneration is influenced by many factors, such as the life history attributes and disturbance of species and the competitive interactions among them. Disturbances play an important role in the regeneration dynamics of many mature hardwood forests [8]–[12]. For pioneer tree species, natural disturbance is regarded as an important measure of population persistence [13]. In China, the natural regeneration of *B. albosinensis* forests in the Qinling Mountains is poor and may soon be replaced by other stable species [5], [14]–[15]. In response to the increasing demand for forest products through regeneration, strip clearcutting was conducted in several *B. albosinensis* forests in the Qinling Mountains as a sustainable alternative for forest regeneration [7], [16]–[17]. Nevertheless, further studies from ecological and silvicultural perspectives on the regeneration dynamics of *B. albosinensis* after strip clearcutting are required.

Understory bamboos in temperate and tropical subalpine forests are particularly effective in reducing seedling recruitment and tree regeneration when they reach a high degree of dominance [18]–[21]. In the Qinling Mountains, *Fargesia qinlingensis* is a common understory bamboo in *B. albosinensis* forests and dominates the understory in most sites. Therefore, understanding the role of *F. qinlingensis* in *B. albosinensis* forests may be critical in determining the regeneration dynamics of the latter. In this study, we analyzed the population structures and spatial patterns of *B. albosinensis* populations in strip clearcut and uncut *B. albosinensis* forests under different bamboo covers. The objectives included the following: (1) identify the regeneration patterns of *B. Albosinensis*, (2) to examine whether *B. albosinensis* regeneration after strip clearcutting was adequate to grow a new forest, and (3) to determine the influence of understory bamboo on the regeneration and community structure of *B. albosinensis*.

## Methods

### Study Area

**Figure 1:**
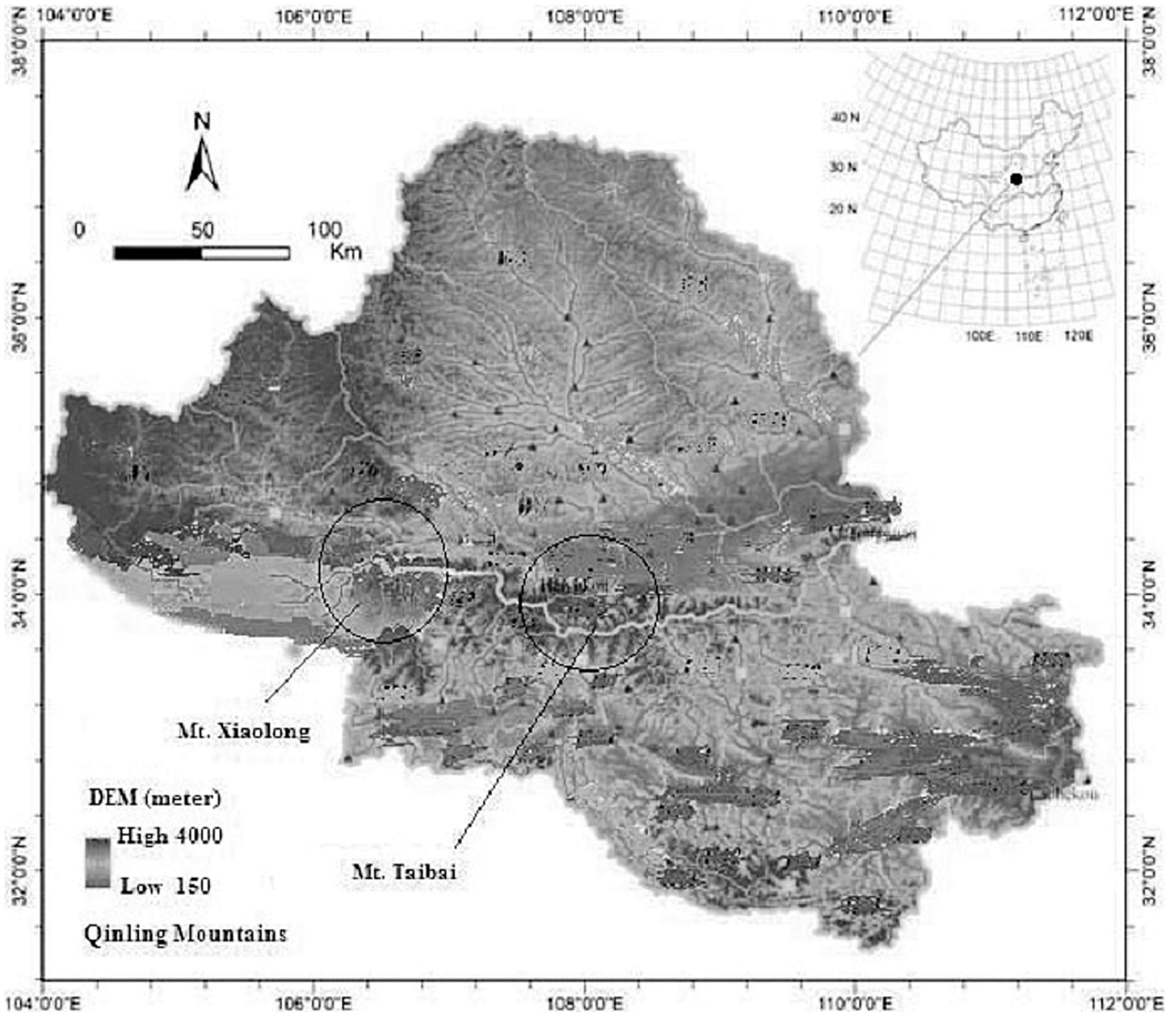
Location of the study areas in the Qinling Mountains of China.

This study was conducted at the Mt. Taibai National Nature Reserve (33°49 to 34°10′N, 107°19′ to 107°58′E, Shaanxi Province) and Mt. Xiaolong National Nature Reserve (33°35′ to 34°06′N, 106°13′ to 106°34′E, Gansu Province), located in the middle and western areas of the Qinling Mountains in China, respectively (Fig. 1). The Qinling Mountains run east–west and act as an important watershed divider between two great rivers of China, the Yangtze River and the Yellow River, which constitute a transitional zone between northern subtropical zone and warm-temperate zone. Mt. Taibai is the highest mountain in the Qinling Mountains, which spans an altitudinal gradient of 530 to 3767 m. Mean annual rainfall is 750 to 1100 mm, primarily falling in June through August, which are also the warmest months with mean monthly temperature of 13.9 and 12.1°C, and December and January are the coldest months with monthly temperature of −5.7 and −4.4°C [15], [22]. Elevation in the Mt. Xiaolong ranges from 704–3300 m. Annual precipitation ranges from 460–850 mm, most of which falls between July and September. Annual mean temperature ranges from 7 to 13°C [2]. The Nature Reserves were established for multiple-uses, including research, animal protection and forest production. Research activities were conducted under the scientific use permits issued respectively by Forestry Department of Shaanxi Province and Forestry Department of Gansu Province. Our field study did not involve any endangered or protected species in the Nature Reserves.

The numbers in parentheses are the estimated seedlings and saplings established in canopy gaps.

*B. albosinensis* forest is an important type of forest vegetation of Qinling Mountains, distributed from 1950 to 2750 m in Mt. Taibai and from 2000 to 2600 m in Mt. Xiaolong. *B. ablosinensis* forests in Mt. Xiaolong were strip clearcut in 1960 s and 1970 s to promote regeneration [2]. In Mt. Taibai, however, human activities are rare due to the relatively difficult accessibility, and some of the only remaining intact forests occur in the region. Thus, the study chooses the stands in Mt. Taibai and Mt. Xiaolong to examine regeneration of *B. ablosinensis* comparatively.

### Field Sampling

After reconnaissance, we selected four cut stands (V–VIII) in the strip clearcut area where the primary trees and undergrowth were felled from the base in the 1960 s, except some large individuals as mother trees. Four stands (I–IV) without cut disturbance were also sampled. Stands were selected if they met the following criteria: (i) *B. albosinensis* dominated the stands and represented the typical forest structure at each site; (ii) the trees in the stands were 100 years, thus classifying them as mature stands; and (iii) sampled stands included observable variations in bamboo cover.

In the current study, each stand was sampled with a large plot (50 m×50 m). Within each plot, all trees with diameter at breast height (or DBH, i.e., 1.3 m above ground level) longer than 5.0 cm were measured. Trees in the stands with DBH less than 5.0 cm and other woody plants with height taller than 1 m were included in five subplots measuring 5 m×5 m, with the trees distributed in the middle and at the four corners of each large plot. Bamboo cover in the stands was estimated by tallying the bamboo coverage in the subplots upon the culms.

### Data Analyses

Woody plant diversity was computed for each stand using Shannon’s formula [23]. Only woody plants that reached 1.0 m in height were considered in the diversity estimation. Species richness in a plot was computed as the number of woody species with stems taller than 1.0 m. Size-structure diagram for *B. albosinensis* was prepared to depict the frequency of different-sized individuals and to interpret the trends in population dynamics.

The spatial pattern of the *B. albosinensis* population was identified using Morisita’s index [24]:
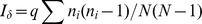
Where *q* is the number of quadrats, *n_i_* is the number of individuals in the *i*th quadrat, and N is the total number of individuals in all quadrats. *I_δ_* equals 1.0 when the population is randomly distributed, *I_δ_* >1 if a population is clumped, and *I_δ_* <1 if a population is regularly distributed. The intensity of pattern was interpreted from the magnitude of the index value. The greater the index value the greater the intensity of clumping. The scale of pattern (m^2^) was identified by computing Morisita’index values for quadrats of varying size. Block sizes were computed using quadrats of 5×5, 5×10, 10×10, 10×15, 15×15, 15×20, 20×20, 20×25, 25×25, and 25×30 units. All of the statistical analyses were conducted using SPSS 11.5 software. Figures were plotted by Origin7.5 software.

## Results

### Stand Characteristics

The basal areas and densities of *B. albosinensis* in different stands varied (Table 1). Basal areas ranged from 18.1 m^2^ ha^−1^ to 22.8 m^2^ ha^−1^ and from 23.8 m^2^ ha^−1^ to 27.6 m^2^ ha^−1^ in the uncut and cut stands, respectively. Densities ranged from 244 stems ha^−1^ to 276 stems ha^−1^ in the uncut stands, whereas *B. albosinensis* were more abundant (range 465 stems ha^−1^ to 693 stems ha^−1^) and had an approximate twofold increase in the cut stands. The bamboo cover ranged from 5% to 50% among the stands. In addition to *F. qinlingensis*, other woody species were also found in *B. albosinensis* forests (Table 2). The other woody plants in the uncut stands were more abundant than those in the cut stands because of strip clearcutting.

**Table 1:** Characteristics of stands and *B. ablosinensis* population in strip clearcut and uncut *B. ablosinensis* forest of Qinling Mountains.

Stands	I	II	III	IV	V	VI	VII	VIII
	Uncut	Uncut	Uncut	Uncut	Strip clearcut	Strip clearcut	Strip clearcut	Strip clearcut
Altitude (m)	2368	2418	2463	2536	2387	2397	2468	2486
Slop (°)	30	28	17	15	32	25	28	26
Aspect	NW	N	NW	N	N	NE	N	W
Bamboo cover (%)	45	15	8	5	5	10	20	50
Density (DBH≧5.0 cm,ha^−1^ )	244	249	267	276	529	693	590	465
Basal area (m^2^ ha ^−1^)	18.1	17.6	22.8	19.3	24.8	24.7	27.6	23.8
Seedlings (<1.5 m tall, ha ^−1^)	16 (11)	8 (6)	16(12)	8 (7)	72	88	58	46
Saplings(<5.0 cm DBH and ≧1.5 mtall, ha ^−1^)	36 (31)	32 (26)	48(39)	28 (24)	106	86	92	76
Observed no. of species	13	16	20	18	15	18	16	11
Shannon diversity (including *F. qinlingensis*)	0.79	1.45	1.63	1.76	1.58	1.68	1.38	0.49

**Table 2:** Number of woody plants (>1.0 m tall) ha^−1^ in the *B.ablosinensis* stands in Qinling Mountains, China.

Speices	I	II	III	IV	V	VI	VII	VIII
	Uncut	Uncut	Uncut	Uncut	Strip clearcut	Strip clearcut	Strip clearcut	Strip clearcut
*Pinus armandii*	48	84	132	104	32	16	28	56
*Betula utilis*		16	48	96	24	56	112	48
*Tilia latevirens*	48	80	46	14	48	32	8	4
*Acer robustum*	56	64	36	48	6	16	32	
*Abies fargesii*			32	82	8	14	84	96
*Ribes glaciale*	68	105	76	38	16	44	80	
*Populus davidiana*		48	38	16	40	16	32	16
*Pterocarya stenoptera*						56	84	16
*Rose omiensis*	96	102	136	12	56	82		30
*Lonicera fangutica*		64	102	44	84	46	34	
*Sorbus discolor*	64	72	84	40				
*Meliosma cuneifolia*		16	32		16	32	64	
*Rhododendron purdomli*		32	64	96				
*Salix matsudana*					32	32	64	
*Salix caprea*	32	48	28	24				
*Cerasus tomentosa*					48	16	32	24
*Populus purdomii*	24	16	32	8	8	16		
*Quercus liaotungensis*	28	32	8					
*Cornus controversa*	24		16				44	16
*Litsea tsinlingensis*					8	16	32	
*Sorbus koehneana*	8	32		6	16		32	
*Acer coesium ssp.giraldii*			8	40				
*Corylus mandshurica*	35	24			8	4		
*Acer miaotaiense*					6		6	34
*Populus purdomii*		8	32	4				
*Acer shenlauensis*				36	12	6	4	
*Ilex yunnanensis*		14	58					
*Pertya sinensis*			6	28				
Total	531	857	1014	736	468	500	772	340
*Fargesia qinlingensis*	1040	790	560	320	470	580	820	1250

Blanks represent no individuals found in the plot.

### Understory Vegetation and Stand Structure

Except the evergreen bamboo (*F. qinlingensis*), deciduous species characterize understory vegetation in terms of number of species (Table 2). Differences in *B. albosinensis* seedling and sapling densities that were observed in the uncut stands seemed were unrelated to bamboo cover (Table 1). In the cut stands, however, *B. albosinensis* density was lower when bamboo coverage in the stands reached 50%. Moreover, seedling and sapling densities were negatively correlated (r = −0.98, p<0.01, Spearman rank correlation coefficient) with bamboo coverage (Table 1). Reduction in other woody plant abundance was also evident when bamboo coverage increased to 50%.

Bamboo density reduced woody plant diversity (Table 1). There was also a negative correlation (r = −0.76, p = 0.032, Spearman rank correlation coefficient) between species richness and bamboo cover. When *F. qinlingensis* densities were included in the Shannon computation, a negative correlation (r = −0.87, p = 0.005, Spearman rank correlation coefficient) was also found between species diversity and bamboo cover (Table 1).

### Size Structure

**Figure 2:**
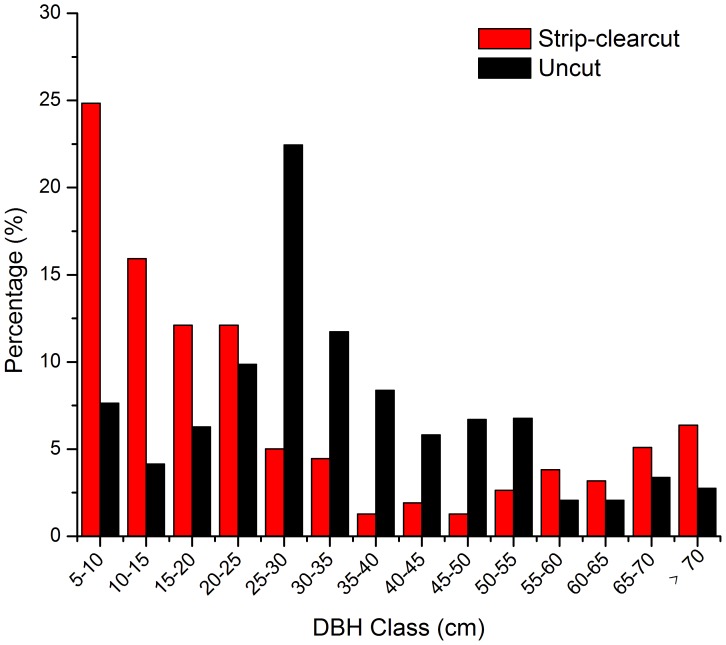
Size class distribution of the *B. ablosinensis* trees in strip clearcut and uncut *B. ablosinensis* forests of Qinling Mountains, China.

The population of *B. albosinensis* showed a bell-shaped diameter distribution pattern in the uncut stands (Figure 2). *B. albosinensis* trees mainly thrived in the middle and larger diameter classes, whereas young *B. albosinensis* with DBH less than 15 cm were scarce and accounted for just 11.9% of the total individuals, suggesting poor recruitment over the past decades. *B. albosinensis* seedlings and saplings were sparser, and only several were found in each uncut stand. The diameter class distribution for *B. albosinensis* in strip clearcut stands had a reverse J shape (Figure 2), with young stems (<20 cm) accounting for 52.8%. Recent recruitment was also abundant, suggesting successful regeneration over the past 50 years after strip clearcutting.

### Spatial Patterns

**Figure 3:**
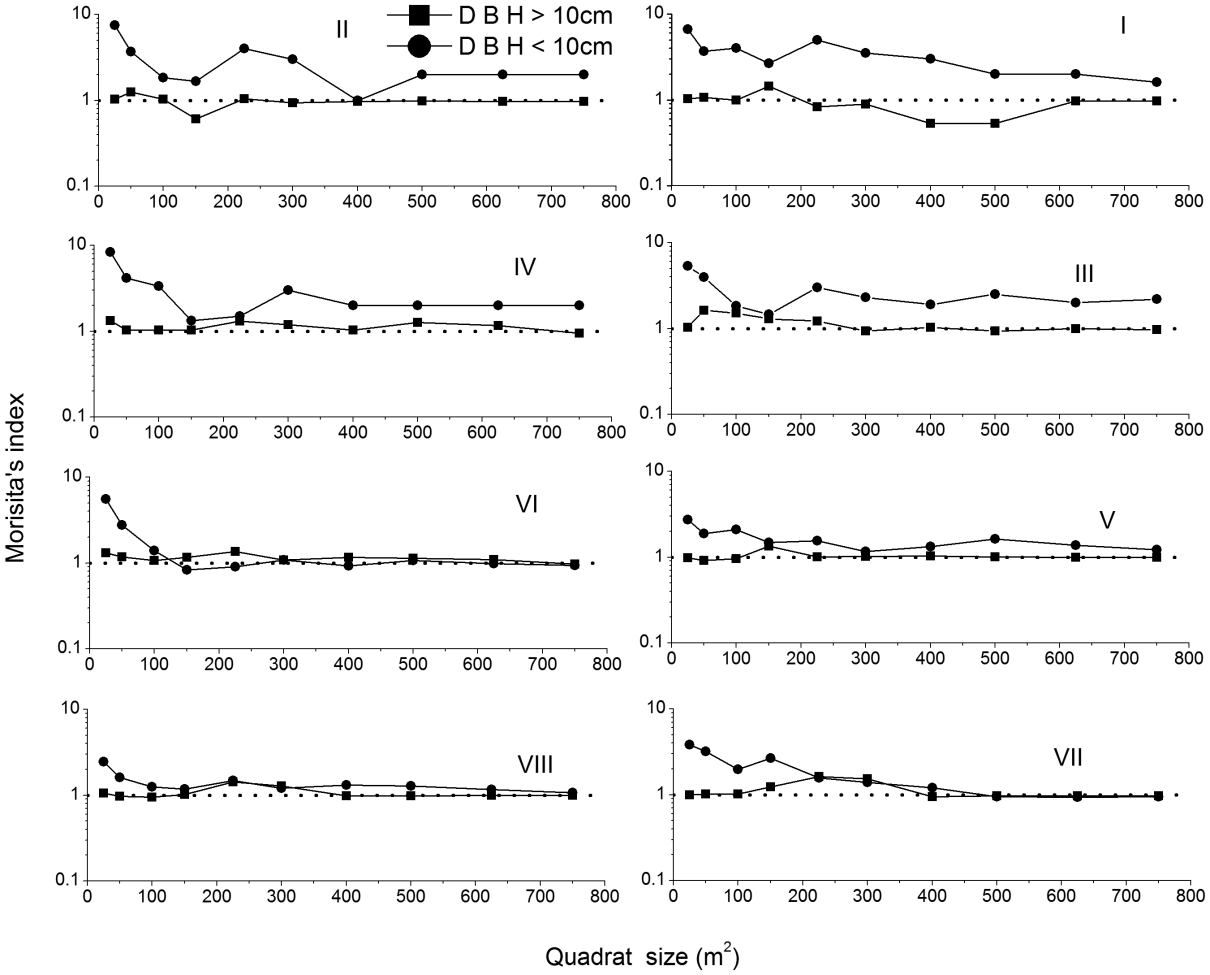
Values of Morisita’s index (*I_δ_*) for different-sized *B. ablosinensis* in 8 stands in Qinling Mountains, China. Random distribution (*I_δ = _*1.0) is shown by the dot line.

Spatial distributions of small (DBH<10 cm) and larger (DBH >10 cm) *B. albosinensis* differed between the cut and the uncut stands (Figure 3). In the uncut stands, small *B. albosinensis* were clumped at almost all scales (25 m^2^ to 750 m^2^), and the clumping magnitude fluctuated severely among different scales, suggesting a heterogeneously natural disturbance. In comparison, small and larger *B. albosinensis* clumps of highest intensity both occurred at small scales (25 m^2^ to 150 m^2^), suggesting that recruitment in single-tree gaps was common. In the cut stands, the high clumping of small *B. albosinensis* occurred at small to intermediate scales, whereas taller *B. albosinensis* trees were clumped only at intermediate scales (200 m^2^ to 400 m^2^). Such results suggested that *B. albosinensis* recruitment in the past period was initiated by large disturbance.

## Discussion

Seedling and sapling pools of *B. albosinensis* in the uncut stands were scarce, suggesting a degeneration trend. Poor natural regeneration of *B. albosinensis* forests elsewhere in China has also been reported [25], [26]. The absence of *B. albosinensis* recruitment generally indicates an unfavorable environment for regeneration. Thick forest litter is a main factor influencing regeneration because of restrictive seed germination in deciduous forests. Based on laboratory simulation experiment results, the germination rate of *B. albosinensis* seeds declined when covered with mulch, especially broad-leaf mulch [6], [7]. Similar effects of forest litter on germination have also been observed in *Betula alleghaniensis* in Canada [27] and *Betula maximowicziuna* in Japan [28]. General field observations demonstrate that *B. albosinensis* are prone to germination in places with less litter fall (e.g., under open-canopy or near roads). Even after a successful germination, *B. albosinensis* seedlings beneath closed canopies easily die as a result of shade sensitivity, although fast-growing seedlings can compensate for less shade-tolerance.

Disturbances drive the regeneration dynamics of most closed-canopy forests by creating opportunities that facilitate the establishment of new individuals through canopy opening [8], [11],[29]. In the present work, poor *B. albosinensis* regeneration was observed in the uncut stands, and these mainly occurred in the canopy gaps produced by disturbances. This finding suggests that *B. albosinensis* are dependent on gaps for regeneration. The intensely clumped distributions of small (<10 cm DBH) and larger (>10 cm DBH) *B. albosinensis* at small scales (25 m^2^ to 150 m^2^) are consistent with the canopy gap sizes (20 m^2^ to 100 m^2^) surveyed in the forests of the Qinling Mountains [4]. Larger *B. albosinensis* were found clumped with lower intensity, indicating a characteristic of thinned population [11]. *B. albosinensis* possess life history traits associated with the gap characteristics, and are less shade-tolerant. In addition, *B. albosinensis* produce more frequent seed crops, have smaller seeds that disperse farther, and have seedlings that grow faster. These life history traits promote the rapid colonization and early dominance of *B. albosinensis* in the gaps. Therefore, gap disturbance seems critical for the maintenance of *B. albosinensis* populations.

However, *B. albosinensis* recruitment in canopy gaps in this report was very limited in number, which may be related to the disturbance scale. *Betula* in other subalpine forests [30]–[32] exhibit great dependency on relatively large gaps for regeneration. *B. albosinensis* also appear to require large gaps for persistence. Natural disturbances that have occurred in *B. albosinensis* forests, including standing death and snapping of canopy trees caused by heavy snow, diseases, and climber twining, are frequently small-scale and rarely large-scale [4], [14]. As a result of these disturbances, light and ground layers are only slightly changed over a small area. Most gaps are filled by either vegetation growth of the surrounding adults or replacement of shade-tolerant species that are already present as suppressed individuals. These frequent small-scale disturbances may not fulfill the demand of *B. albosinensis* regeneration for an environment with much light and exposed soil. Therefore, without large-scale disturbances to clear space and expose the covered land, it may be difficult for *B. albosinensis* stands to persist for generations to come.

Bamboo, as a common understory plant in subalpine forests, restricts tree regeneration and species diversity [20]–[21], [29], [33]. Where bamboo fully occupies a forest understory, the frequency and number of tree seedlings and shrubs are lower because of intense competition with bamboo for space and resources. In our study, a significant negative correlation was found between understory woody plant diversity (richness and Shannon’s formula) and *F. qinlingensis* cover, suggesting that dense bamboo reduced plant diversity. This finding is consistent with published reports on other subalpine forests where bamboos dominate the forest understory [18], [34], [35]–[37]. However, no significant correlation was found between *B. albosinensis* recruitments and *F. qinlingensis* cover despite the wide range (5% to 45%) in the uncut stands. Previous studies in mixed hardwood-conifer forests have suggested that understory bamboos impede *Betula* regeneration, although it did so with less intensity than conifer [36], [37].

It is likely that there are other restricting factors (e.g., forest litter, closed canopy, and dense shrubs) that weaken the impeding effect of bamboo alone on *B. albosinensis* regeneration. In addition, the ability of *B. albosinensis* to disperse into gaps as well as go through a fast-growing juvenile stage may more or less help them break the shade of bamboo layers. Therefore, bamboo density has no significant effect on the distribution and establishment of *B. albosinensis* seedlings and saplings. However, understory bamboo may contribute greatly to the persistence of *B. albosinensis* population by restricting coexisting conifer species [32], [37]. Furthermore, bamboo flowering usually creates a favorable environment for *B. albosinensis* regeneration as a large forest disturbance.

Contrary to failed natural regeneration, a large number of recruitments were found in stands with subsequent regeneration approximately 50 years after strip clearcutting. *B. albosinensis* dominated the regenerating stands as a pioneer species. After strip clearcutting, the sudden exposure of previously forested lands and more sunlight reaching the forest floor seemed responsible for the successful regeneration of sunlight-loving *B. albosinensis.* The establishment of *B. albosinensis* in large numbers after strip clearcutting suggests that disturbance magnitude is important for understanding the regeneration strategy of *B. albosinensis.* However, we found that high bamboo coverage in the cut stands reduced *B. albosinensis* seedling and sapling recruitment. With vegetation restoration after the cut, *B. albosinensis* regeneration became prone to bamboo restriction when they attained a high degree of dominance. As such, other artificial silvicultural methods, such as bamboo removal, may be necessary in improving the competitive status of *B. albosinensis* at the later stage of forest regeneration after clearcutting.

The above results confirm that strip clearcutting can prevent primary *B. albosinensis* stands from degenerating. The moderate severity of disturbance caused by strip clearcutting may be the best approach to achieve the dual objectives of forest conservation and timber production. Palynological evidence suggests that *Betula* forests have existed as zonal forests in the geological period and in modern times in the Qinling Mountains [38]. Naturally, *B. albosinensis* population may be capable of maintaining their stability as a whole despite the particularly poor regeneration. Thus, the aggregation of different-spatiotemporal cohorts driven by disturbances may be the pattern and strategy of natural *B. albosinensis* population stability. However, persistent and periodic artificial disturbance by strip clearcutting is necessary from a forest production perspective.
